# Research protocol for the Paraesophageal hernia symptom tool, a prospective multi-center cohort study to identify the need and threshold for surgery and assess the symptom response to surgery

**DOI:** 10.1093/dote/doad028

**Published:** 2023-05-08

**Authors:** Nainika Menon, Nadia Guidozzi, Swathikan Chidambaram, Aiysha Puri, Viknesh Sounderajah, Lorenzo Ferri, Ewen A Griffiths, Donald Low, Nick Maynard, Carmen Mueller, Manuel Pera, Mark I van Berge Henegouwen, David I Watson, Giovanni Zaininotto, George B Hanna, Sheraz R Markar

**Affiliations:** Department of general surgery, Oxford University Hospitals National Health Service Foundation Trust, Oxford, UK; Department of General Surgery, University of Witwatersrand, Johannesburg, South Africa; Department of Surgery and Cancer, Imperial College London, St Mary’s Hospital, London, UK; Department of Surgery and Cancer, Imperial College London, St Mary’s Hospital, London, UK; Department of Surgery and Cancer, Imperial College London, St Mary’s Hospital, London, UK; Department of Surgery, McGill University Health Centre, Montreal, Quebec, Canada; Department of Surgery, Univeristy of Birmingham National Health Service Trust, Birmingham, UK; Department of Thoracic Surgery and Thoracic Oncology, Virginia Mason Medical Centre, Seattle, Washington, USA; Department of Surgery, Oxford Upper GI Centre, Churchill Hospital, Oxford University Hospitals National Health Service Foundation Trust, Oxford, UK; Department of Thoracic Surgery and Thoracic Oncology, Virginia Mason Medical Centre, Seattle, Washington, USA; Section of Gastrointestinal Surgery, Department of Surgery, Hospital del Mar Medical Research Institute, Barcelona, Spain; Division of Surgery, UMC Amsterdam, Amsterdam; Department of Surgery, College of Medicine and Public Health, Flinders University, Adelaide, Australia; Department of Surgery, Hospital SS Giovanni e Paolo, Venezia, Italy; Department of Surgery and Cancer, Imperial College London, St Mary’s Hospital, London, UK; Department of general surgery, Oxford University Hospitals National Health Service Foundation Trust, Oxford, UK

**Keywords:** hernia, paraesophageal, surveys and questionnaires

## Abstract

Large hiatus hernias with a significant paraesophageal component (types II–IV) have a range of insidious symptoms. Management of symptomatic hernias includes conservative treatment or surgery. Currently, there is no paraesophageal hernia disease-specific symptom questionnaire. As a result, many clinicians rely on the health-related quality of life questionnaires designed for gastro-esophageal reflux disease (GORD) to assess patients with hiatal hernias pre- and postoperatively. In view of this, a paraesophageal hernia symptom tool (POST) was designed. This POST questionnaire now requires validation and assessment of clinical utility. Twenty-one international sites will recruit patients with paraesophageal hernias to complete a series of questionnaires over a five-year period. There will be two cohorts of patients—patients with paraesophageal hernias undergoing surgery and patients managed conservatively. Patients are required to complete a validated GORD-HRQL, POST questionnaire, and satisfaction questionnaire preoperatively. Surgical cohorts will also complete questionnaires postoperatively at 4–6 weeks, 6 months, 12 months, and then annually for a total of 5 years. Conservatively managed patients will repeat questionnaires at 1 year. The first set of results will be released after 1 year with complete data published after a 5-year follow-up. The main results of the study will be patient’s acceptance of the POST tool, clinical utility of the tool, assessment of the threshold for surgery, and patient symptom response to surgery. The study will validate the POST questionnaire and identify the relevance of the questionnaire in routine management of paraesophageal hernias.

## INTRODUCTION

Hiatal hernias can be classified into four types, the most common of which is a type I hiatal hernia which represents a sliding hernia. Type II hernias are otherwise known as paraesophageal hiatal hernias—they do not have a sliding component and the gastro-esophageal junction remains anatomically below the diaphragm. A type III hernia is a combination of types I and II (sometimes called a mixed hernia), whereas a type IV hernia contains the stomach and additional abdominal viscera in the hernia sac.^1^^,^^2^

Treatment options for large hernias, usually containing 50% + of the stomach within them, and with a significant paraesophageal component (types II, III, IV) include conservative management or surgical intervention. One of the rare risks of the former, when large hernias are present, is that of strangulation or incarceration, which may ultimately lead to an emergency paraesophageal hernia repair which has a poorer prognosis and outcome.^1^ Furthermore, and perhaps more importantly, large paraesophageal hernia risk continued enlargement over time with a possible increase in symptoms. Elective surgical options include minimally invasive or open repairs which are often technically challenging in a typically elderly, co-morbid patient cohort.

There are a wide range of insidious symptoms that may be associated with paraesophageal hernias, such as weight loss, dysphagia, dyspnea, shortness of breath, heartburn, chest pain, hoarseness of voice, early satiety and anemia.^3–5^ The prevalence of upper gastrointestinal symptoms in paraesophageal hernias provides the rationale for using health-related quality of life (HRQoL) questionnaires focusing on gastro-esophageal reflux disease (GORD) to assess patients before and after surgery.^6–8^ As the questionnaires are focused on GORD symptoms alone, they do not take into consideration the other symptoms that are associated with paraesophageal hernias. The symptoms that are not included in the current HRQoL tools are sometimes the very reason patients seek surgical intervention.^6–8^

Additionally, the current screening questionnaires do not fully identify a patient’s motivations for surgical repair, which often relates to their broad symptoms and its impact on their quality of life. For those who undergo surgical repair, the symptom response following surgery has not been thoroughly studied.

Due to the lack of paraesophageal hernia screening tools, this study group devised a disease-specific questionnaire (POST questionnaire) in 4 stages; first a Steering Committee was formed, followed by a systematic review and online scoping survey and then a Delphi consensus. The final stage consisted of two international patient workshops to assess the acceptability and usability of the tool.^9^

The aim of this study is to assess the clinical utility and longitudinally validate a paraesophageal hernia symptom tool (POST)^9^^,^^10^ for the clinical assessment of patients with paraesophageal hernias—types II to IV. The study will test POST in patients before paraesophageal hernia repairs to assess the need and threshold for surgery, and it will be used in patients before and after paraesophageal hernia repairs to assess the symptom response to surgery.

## OBJECTIVES

### Global objective

- To assess the clinical utility of the POST tool for the clinical assessment of patients with paraesophageal hernias—types II to IV

### Specific objectives

- Patient’s acceptance of the POST testing tool
- Beta test POST in patients before paraesophageal hernia repair to assess the need and threshold for surgery
- Beta test POST in patients before and after paraesophageal hernia repair to assess the symptom response to surgery

## METHODS

A total of 21 esophago-gastric units internationally will be invited to participate as participant identification centers (PIC). Given the caseload in each center, we are aiming to recruit approximately 500 patients. The centers will be asked to recruit patients being assessed for primary paraesophageal hernia repair over a 24-month study period as per the inclusion and exclusion criteria.

### Inclusion criteria

- Age over 18 years old
- All type II-IV hernias with a paraesophageal component confirmed on computerized tomography (CT) or endoscopy or barium study
- Able to complete the POST questionnaire (hybrid model—patient-led or clinician-led depending on center preference

### Exclusion criteria

- Unable to provide informed consent
- Previous paraesophageal hernia repair or esophagi-gastric surgery
- Diagnosis of an esophagi-gastric cancer
- Type 1 sliding hiatus hernia
- Emergency paraesophageal hernia at presentation/surgery

There will be two cohorts of patients fulfilling the study criteria—1. Patients undergoing surgical management of paraesophageal hernias and 2. Patients managed conservatively (observational cohort). All patients will be followed up for 1 year. The surgical cohort will be followed up for a total of 5 years postoperatively to assess for symptomatic disease recurrence.

Appendices 1 and 2 summarize the key time points for the study. When a patient consents to participate in the study, they will be asked to complete the following questionnaires: 1. Validated quality of life tool (GORD-HRQL) which provides a measure of current best practice for assessment of health-related quality of life (Appendix 3) 2. POST questionnaire (Appendix 3) and 3. Satisfaction questionnaire regarding the use of POST for symptom assessment. Some centers may have long waiting times between the first clinical review and the date of surgery. For those who are operated on within a year of their first clinical review, the baseline questionnaires listed above will be completed in clinic. For patients who are operated on more than a year after their first clinical review, the baseline questionnaires will be repeated within a 2-week period prior to surgery to capture any change in symptoms. Additional investigations that were performed during this prolonged waiting time will also be recorded.

In order to ensure patient inclusivity, the questionnaires will be translated into English, Italian, French, Spanish, and Dutch to cover the native languages at each study center.

Patient demographics, clinical data, results of paraesophageal hernia investigations and clinical outcome data from surgery including recurrence over the 5 year follow-up period, will also be collected. There is accepted variation in the preoperative and postoperative investigations including CT scans, barium swallow, endoscopy, and pH/manometry testing. All data will be stored on the RedCap online database.

The POST platform will be made into an online web or app-based tool using Qualtrics. This will allow patients to complete the questionnaire either in a clinic environment, via telephone or at home. An alternative paper version will also be made available for completion and correspondence via post. Given the international variation in completing these questionnaires by patients, some centers may offer clinician-led telephonic/face-to-face completion of the questionnaire, accepting the potential reporting bias. If a center has long waiting times between the first clinical review and the date of surgery, the preoperative questionnaires can be completed closer to the date of surgery or on the day of surgery.

Those who undergo surgery to repair their paraesophageal hernias will be asked to complete these questionnaires again at the following postoperative time points: 4–6 weeks, 6 months, 12 months and then annually for a total of 5 years postoperatively. The conservatively managed cohort will also be asked to repeat the questionnaires at 1 year to follow up to assess for symptom progression and whether there was a change in the decision for surgery. Given the variation in follow-up protocols and investigations between different centers, each center will follow their standard practice and outline the details of this in their data. The two questionnaires (1. Validated quality of life tool (GORD-HRQL)^11^ which provides a measure of current best practice for assessment of health-related quality of life and 2. POST questionnaire) will be completed at each time point. The satisfaction questionnaire regarding the use of POST for symptom assessment will only be used for the first year following the operation. Follow up in the form of electronic, telephone or paper-based questionnaires will be conducted by each center for the first year. Subsequent annual follow-up will be conducted either by the center or the POST research team. All results will then be uploaded onto the Redcap database, either by the local center or by the study organizers.

## SAMPLE SIZE

Sample size will be dependent on how many patients consent to be enrolled in the study from the 21 sites, over the 24-month recruitment period. This decision was based on the variability in the caseload between different centers. The predicted sample size will be 500 patients.

## ETHICS

Ethics approval is pending from the Research Ethics Committee (REC) and Health Research Authority (HRA) for the United Kingdom arm of the study. Ethics approval will be obtained from the appropriate local ethics department at each individual site involved in the study. The study will be conducted in accordance with the recommendations for physicians involved in research on human subjects adopted by the 18th World Medical Assembly, Helsinki 1964, and later revisions.

No interim data will be analyzed. Data will only be analyzed at the end of the 24 month recruitment period and then for a total of 5 years in order to review the long-term outcomes.

All patients will complete a written consent form and consent to their email, telephone numbers and postal address being used for disseminating the questionnaires. The chief investigator will be responsible for data protection. Data will be stored in a secure environment under password protection where study personnel will have exclusive access. Every effort will be made to keep patient information anonymous and all data will be destroyed after 10 years.

## DISSEMINATION AND DELIVERABLES

The findings of this study will be shared internationally through various modalities including publication in a high impact clinical journal and presentations at surgical and gastroenterology meetings. The first set of data will be released at the one-year timepoint. The validated tool will be shared with patients and surgeons with a view to implement this tool in routine paraesophageal hernia management.

### Collaborators

R. Aye, B. Louie (Swedish Cancer Institute and Medical Centre, Seattle, USA); R. Baigrie (Kingsbury Hospital and Groote Schuur Hospital, Cape Town, South Africa); L. Bonavina (University of Milan, Milan, Italy); G. Darling (Toronto General Hospital, Toronto, Canada); P.M. Fisichella (Northwestern University Feinberg School of Medicine, Chicago, USA); S. Jaume-Bottcher (University Hospital del Mar, Barcelona, Spain); J.C. Lipham (University of Southern California, Los Angeles, USA); W.S. Melvin (Montefiore Medical Centre, New York, USA); K. Nason (University of Massachusetts Chan Medical School, Springfield, USA); B. Oelschlager (University of Washington, Seattle, USA); F. Puccetti, R. Rosati (San Raffaele Hospital, Milan, Italy); J.S. Roth (University of Kentucky, Lexington, USA); P. Siersma (University Medical Centre Utrecht, Utrecht, The Netherlands); B. Smithers (University of Queensland, Woolloongabba, Australia); N. Soper (University of Arizona College of Medicine, Phoenix, USA); S. Thompson (Flinders University, Adelaide, Australia).

## Data Availability

Data sharing is not applicable to this article as no new data were created or analyzed in this protocol paper. However, the study will generate data that support the findings of this study and will be available from the corresponding author, SRM, upon reasonable request.

## Notes

Financial support: None.

Potential competing interests: None.

## Appendices

### Appendix 1. Data collection flowchart

**Appendix 2:** Summary of investigations, treatment, and assessments

Exam	Pre-op		Post-op						
	At diagnosis	Within 2 weeks of surgery (only if surgery is more than a year after the first clinical review)	4–6 weeks	6 months	12 months	2 years	3 years	4 years	5 years
CT scan/barium swallow/endoscopy/ph studies/manometry	X					+/−	+/−	+/−	+/−
History, physical exam	X								
Informed consent	X								
POST evaluation form	X	X	X	X	X (operative and conservative cohorts)				
GORD-HRQL	X	X	X	X	X	X	X	X	X
POST	X	X	X	X	X	X	X	X	X

**Appendix 3:** GORD-HRQL questionnaire

Symptoms	No symptoms = 0 Symptoms noticeable but not bothersome = 1 Symptoms noticeable and bothersome, but not everyday = 2 Symptoms bothersome everyday = 3 Symptoms affect daily activities = 4 Symptoms are incapacitating, unable to do daily activity = 5					
How bad is your heartburn?	0	1	2	3	4	5
Heartburn when lying down?						
Heartburn when standing up?						
Heartburn after meals?						
Does heartburn change your diet?						
Does heartburn wake you up from sleep?						
Do you have difficulty swallowing?						
Do you have pain with swallowing?						
Do you bloating or gassy feelings?						
If you take medication, does this affect your daily life?						
How satisfied are you with your present condition?	Satisfied Neutral Dissatisfied					

**Appendix 3:** POST questionnaire

Symptom	Do you have any of the following symptoms and how often?				How much impact does the symptom have on your life? (0 = none; 5 = severe)					
	Never 0	Monthly 1	Weekly 2	Daily/multiple times per day 3	0	1	2	3	4	5
Difficulty getting solid foods down										
Difficulty getting liquids down										
Chest pain after meals										
Shortness of breath after meals										
Early feeling of fullness after eating										
Heartburn or reflux										
